# “Killing in the Name of 3R?” The Ethics of Death in Animal Research

**DOI:** 10.1007/s10806-024-09936-y

**Published:** 2024-12-02

**Authors:** Kirsten Persson, Christian Rodriguez Perez, Edwin Louis-Maerten, Nico Müller, David Shaw

**Affiliations:** 1 Institute for Biomedical Ethics, University of Basel, Basel, Switzerland; 2 Institute for Animal Hygiene, Animal Welfare, and Farm Animal Behaviour, University of Veterinary Medicine, Hannover, Foundation, Hanover, Germany; 3 Philosophical Seminar, Department of Arts, Media, Philosophy, University of Basel, Basel, Switzerland; 4 Care and Public Health Research Institute Maastricht University, Maastricht, Netherlands

**Keywords:** Animal research, Animal ethics, Animal death, Killing animals, 3R, Animal welfare law

## Abstract

Changing relationships with nonhuman animals have led to important modifications in animal welfare legislations, including the protection of animal life. However, animal research regulations are largely based on welfarist assumptions, neglecting the idea that death can constitute a harm to animals. In this article, four different cases of killing animals in research contexts are identified and discussed against the background of philosophical, societal, and scientific-practical discourses: 1. Animals killed during experimentation, 2. Animals killed before research, 3. “Surplus” animals and 4. “Leftover” animals. The scientific community and, accordingly, animal research regulations such as the internationally acknowledged framework 3R (“Replace”, “Reduce”, “Refine”) tend to aim at the reduction of “surplus” and, to some extent, “leftover” animals, whereas the first two classes are rather neglected. However, the perspective that animal death matters morally is supported by both societal moral intuitions and certain theoretical accounts in animal ethics. Therefore, we suggest the implementation of the 3Rs in regulations, so that they: 1. Make their underlying philosophical position transparent; 2. Are based on a weighing account of animal death; 3. Are applicable to procedures on living and dead animals; 4. Apply the “reduction” principle to procedures on dead animals; 5. Entail that methods using (parts of) dead animals need to be replaced by animal free methods, if possible; 6. Do not suggest replacing research on living animals by research on killed animals; 7. Include all kinds of animals, depending on the respective harm of death; 8. Are applied to the broader context of experimentation, including breeding and the fate of the animals after the experiment.

## Background

In the past decades, changing relationships with nonhuman animals (from here, “animals” for better readability) are reflected in important modifications in animal welfare legislations in different European countries. Still, most European animal welfare laws are focusing on the sentientist goal to prevent those animals who are capable of suffering and feeling pain from doing so in cases of human-animal interaction. Groups of animals who, according to more recent research, share those properties and are, therefore, considered by the law, have been continuously added (Fiorito et al., 2014). The idea that there can be some way of affecting animals in a morally relevant way beyond inflicting pain or suffering led to recent developments in animal welfare legislation, e.g., the inclusion of “intrinsic value” and “integrity” in the Dutch Animal Welfare Act (§1, article 1.3) or the “dignity of creature” in the Swiss one (§1, article 3 a.). Furthermore, there is an ongoing debate in society as well as in philosophy to what extent animal lives per se should be protected and whether (even painless) death means morally relevant harm to the individual animal, as we will point out in section “Death as a Harm in Animal Ethics”. This phenomenon is reflected by the demand for a “reasonable cause” for killing animals in the German legislation (§1 German Animal Welfare Act); or the status as a “fellow creature” in the Austrian law (§1 Austrian Animal Welfare Act).

When it comes to laws and guidelines for animal experimentation, explicit and detailed regulations are still focused on the sentientist conception of preventing pain, suffering, and harm “on live animals” (EU directive 2010/63, article 10). Painless killing of animals in experiments and defining humane endpoints in order to prevent further suffering and pain are core elements of many European animal experimentation guidelines, including the EU directive 2010/63, the Animal Scientific Procedures Act 1986 in the UK, or the internationally acknowledged 3R principles (Replace, Reduce, Refine).

The literature provides, on the one hand, some discussion on the legal justification for killing animals for animal experiments, including the demand for further research in this area (Chmielewska et al., 2015) and, on the other hand, some suggestions how to extend the 3Rs with more “Rs” to avoid killing animals that are not or no longer used for animal experimentation (Franco, 2016). What is missing is a comprehensive consideration of all cases of killing related to animal experimentation, a differentiation of philosophical arguments emerging from the corresponding debates, and a suggestion of how to include “death as a harm” in European animal experiment regulations and guidelines, particularly in the 3Rs.

We will begin with a classification of the cases of killing animals in the context of animal experimentation and an illustration of the scientific perspectives on those cases which will then relate to the (European) societal perceptions of death as a harm for nonhuman animals as well as the philosophical debate on animal death and euthanasia. In a second step, we want to investigate potential relations of the classifications pointed out in findings of the first step to the 3Rs (as originally implied by Russell and Burch and as implemented in science today).

This research is part of the Swiss National Research Programme 79 on “Advancing 3R—Animals, Research and Society” [5] and, thus, aims at contributing to the revision of 3R as a framework for animal research in Switzerland, including twenty-first century perspectives on human-animal relationships. Accordingly, we conclude by suggesting a revision of some of the basic assumptions of 3R in order to update guidelines regulating animal experimentation in Switzerland and the EU regarding the value of animal life and death.

## Killing Animals in Research

### Cases of Killing Animals in Research

Killing is the infliction of death. This definition may seem trivial, but it has two crucial upshots: First, not every dead animal has been killed. In the context of laboratory research, even more cases would have to be listed, namely those animals who die in breeding facilities or during the transport from there to the laboratory; while being housed in the laboratory or during and after experimental procedures. While the consequences for the animals may be similar, there is a huge ethical and legal difference between killing animals and coping with animal death as an unintended event. In this article, we, as bioethicists, want to focus on action-guiding principles, and, accordingly, on killings in the above-defined sense, i.e. decisions and actions that can be regulated. Second, the above definition of killing implies that “killing” is intended as a purely descriptive term that carries no moral evaluation. This sets it apart from the negative “murder” on the one hand and the positive “sacrifice” on the other.

Furthermore, there are cases of regulatory killing which will not be dealt with in this article, even though they happen in the context of laboratory research, e.g., killings if studies are suspended for some reasons, if there are disease outbreaks in a laboratory stable or if the risk of zoonotic infections is too high. These killings are done for legal reasons beyond the influence of experimental design and decision-making in the laboratory, and, accordingly, beyond the scope for the 3Rs. Therefore, we consider them not within the scope of our considerations, even though, for example, institutionalising a culture of care could promote the reduction of those animal deaths.

From a technical point of view, there are at least four different forms of killing animals (as an action inflicting death) in laboratory research, see Table 1.

**Table 1:** Four classes of killing in animal research

(a) Killed during experimentation	Animals -Are part of an approved study, including the definition of “humane endpoints” -At least *prima facie*—killed by euthanasia in the narrow sense of euthanasia, i.e., killed *lege artis* in the animals’ own interest at that moment (Persson et al., 2020), i.e., suffering or harm would be unbearable if they lived on -Or killed by other methods (e.g., slaughter or decapitation (Agbeniga et al., 2013; Palo et al., 2013)) at a pre-determined point of the experiment because, e.g., the research plan includes taking their organs, tissue, or a large proportion of their blood
(b) Killed in advance	Animals -Are killed before research is done with their bodies. Legally speaking, no animal experimentation (see, e.g., (Campagnol, 2022, p. 252)); different rules for approval/ no need to consult animal research commissions -Killing method is dependent on animal welfare-related criteria and research design; e.g., histopathological changes to organs rendering them unsuitable for investigation when killed with euthanizing agents (Feldman & Gupta, 1976), thus a physical method (e.g. slaughter or decapitation) would be preferable
(c) “Surplus”	Animals are bred in the context of experimentations but then not used for experiments -Because only one sex is used, -Because they do not carry the phenotypical properties that are present in only some percentage of a litter, -Because they are just bred to provide company of other laboratory animals, -Because a pool of individuals is needed to keep a breeding line intact, -Because the exact number of animals that is born cannot be known and some backup is always calculated but not used -For logistic and economic reasons, these animals cannot be taken care of until they die of other causes, which is why they are killed
(d) “Leftovers”	Animals that were -Used for an experiment during which a humane endpoint prescribing killing was not reached -Do not usually suffer from pain but can no longer be used for experiments due to their past experiences -Killed for the same reason as the “surplus” animals

In all four cases, the killing of vertebrate and some invertebrate species is legally regulated. In the EU directive 2010/63, for example, Annex IV provides a specific list of methods for each vertebrate group. Only trained persons are authorised to perform the killing and it should not take place in areas where other animals are present.

The numbers of animals that can be classified as one of these four cases are documented and reported to different extents. In Switzerland and the EU, all animals that were mentioned in a project application and approved by an animal experimentation commission, that is animals killed during experimentation and “leftovers”, are documented and published. See, e.g., BLV (2022). Additionally, according to the EU directive 2010/63, all member states must report the numbers of “surplus” animals that are bred and killed but not used in research (used to obtain organs or tissues, used for breeding but no longer sufficiently productive, those that fell sick before they could be used) every five years. In 2017 that was the case for more than 12 million animals, mostly mice (83%) and zebrafish (7%) (European Commission, 2020). For Switzerland, the numbers of animals who are bred and killed but not used are not reported. However, the Swiss Federal Food Safety and Veterinary Office (FSVO) states that the vast majority of the animals not used in experiments are killed (FSVO, 2023).

### Cases of Killing and the 3Rs

In their “Principles of Humane Experimental Technique”, Russell and Burch aim at the “removal of inhumanity” (Russell & Burch, 1959, p. 66) towards animals in experimental research. By the technical term “inhumanity” they refer to distress, including pain, fear, and further unpleasant states. When writing about “contingent inhumanity” they elaborate on that kind of animal death they call “contingent mortality” which is “not part of the experimental intention” (Russell & Burch, 1959, p. 55). Rather in line with the recent legal development of the EU directive 2010/63, Russell and Burch tried to collect data on different groups of laboratory animals who died unintendedly, such as guinea pigs who had served as “stock animals (unused)” or mice who had “died on account of toxic urines” (Russell & Burch, 1959, p. 56).

By comparing the numbers of animals that accidentally die during experiments and the death of “unused” animals, Russell and Burch come to the conclusion that for most experimental procedures, the animals’ deaths “can, therefore, be ascribed to very slight defect in husbandry, rather than in the experimental procedure itself” (Russell & Burch, 1959, p. 56). Additionally, they provide examples for procedures that reduce the mortality in experiments (ibid) and call it “the removal of special contingent inhumanity”.

When it comes to the (action-guiding) 3Rs, these do not serve the purpose of protecting the animals’ lives but their welfare (in the sense of absence of strain, see (Rodriguez Perez et al., 2023)). As far as it concerns welfare, killing is regulated in terms of refinement, for example, in the EU directive 2010/63: “among the methods available, only those including “a good death” should be used (humane killing)” (Campagnol, 2022, p. 252). Besides the point that the option of painless killing may not always be given—if (painless) death is not a welfare issue, the 3Rs are not directed at preventing animals from being (painlessly) killed (even though painless killing presents a challenge in itself).

While Russell and Burch did not elaborate on their moral views about animal death, their employer and supervisor, Charles W. Hume, explicitly endorsed a welfarist view of animal death, stating: “There is no harm in killing animals provided it be done painlessly” (Hume, 1962) (p. 130, similarly p. 191). Hume also endorsed a version of the replaceability argument (see Sect. 2.3), arguing that “to kill an important being is a more serious matter than to kill an unimportant one […]. Human beings are more important than animals, and it is a much more serious thing to kill a man than to kill an animal.” (Hume, 1956) (p. 51). The original philosophy behind the 3Rs was thus strongly committed to the view that killing animals is not morally problematic.

How do the 3Rs, accordingly, relate to the categories of animals killed in research?

(a) Killed during experimentation.

By the application of “Reduction” and “Replacement”, science indirectly already aims at a decrease in the number of animals who are killed during or immediately after experiments. However, this is just a side effect. 3R implementations such as the EU Directive 2010/63 are directed at lowering the number of and, in the end, fully replace experiments on living animals, and eliminating or reducing the amount of pain, suffering, distress, or lasting harm (see Art. 4). Painless killing is considered a refined method as it does not cause (but end) further pain, suffering, or distress.

(b) Killed in advance.

The 3Rs are not applied to this kind of research if it is not defined as animal experimentation. Both the EU Directive and the Swiss law explicitly define animal experiments as procedures that include the use of living animals (EU Directive 2010/63, Art. 4, Swiss Animal Welfare Act, Art. 3). In the Swiss case, a simplified animal experimentation licensing procedure is still required to experiment on dead animals, but such cases are considered as “severity degree 0″ and therefore are not, in principle, a concern for the 3Rs. In any case, counting the use of previously killed animals as Replacement seems to “sell” the procedure and it might be perceived in a wrong way by parts of the public if it is the case that death is considered a harm to animals in some societal, veterinary or philosophical discourses. This, however, only applies to animals that are killed in advance for the particular research purpose. In other cases, animals might be used that were killed for other reasons (e.g., as “surplus” or “leftover” animals or during an experiment). That process can indeed be considered a reduction as fewer animals are killed (they were dead anyway) but it is logistically demanding. Institutions or organisations can provide detailed databases on the dead animals that are killed in some experiments and stored to make them available for other (intern or extern) working groups, see, e.g., “animatch” (AniMatch UG). As long as killing is considered”no harm”, all alternatives, even if only involving mild harm, should be considered worse. In case killing was considered a harmful procedure (severity degree > 0), however, the harmfulness of alternatives would have to be compared in a weighing process.

(c) and (d) “Surplus” and “leftover”.

“Surplus” and “leftover” animals are not directly included in the 3Rs. Even though the 3Rs—as they are applied today—are occasionally extended to the contexts of preparation, supply, “waste”, logistics etc. (see, e.g. (Swiss 3R Competence Centre [3RCC]), they are directed at protecting animal welfare rather than animal life per se.

However, some implementations of the 3Rs suggest that the use of animal-free research would or should be preferred even over research on animals that does not cause welfare issues but the animals’ death, see, e.g. the 3Rs definitions according to the NCRS (The 3Rs | NC3Rs). Furthermore, guidelines such as the British Guidance on the operations of the Animals (Scientific Procedures) Act 1986 demand in their section “The 3Rs and choice of methods” a minimisation of surplus breeding.

### Death as a Harm in Animal Ethics

In animal ethics, there are different viewpoints regarding the question in what way and to what extent death means a harm to animals. There is no general agreement among philosophers on the moral relevance of animal death, and, accordingly, no univocal suggestion how to weigh killing animals as a morally relevant action. There are, roughly speaking, two clusters of approaches towards that question.

(1) Death does not matter morally

Following an animal welfarist account, a sentient animal that is not (to a sufficient extent) self-conscious cannot envision themselves in a future situation and, accordingly, has no desire to live on (DeGrazia, 1996, p. 235). One argument resulting from this account of animal death is the replaceability argument. According to this argument, famously defended by Peter Singer (Singer, 2011), the death of a sentient being that lacks certain cognitive capacities does not matter as long as it can be replaced by another being of that kind. As long as the next individual reaches the same amount of overall well-being, therefore, it is not morally relevant if the former individual is killed. Two premises which are relevant in the context of animal experimentation, are combined in this argument.

- Animal welfare is not to be evaluated strictly for each individual but rather to be summed up in total (“total utilitarianism”).
- Animal death is not considered a harm to most animals (i.e., according to Singer, those beings who are “conscious, but not self-conscious […]. They will not have desires that project their images of their own existence into the future.” (Singer, 2011, p. 126).

For animal experimentation this approach seems to be important when comparing potential experimental designs regarding the number of suffering animals against the severity of suffering (Tannenbaum & Bennet, 2015). If painless killing is classified as “no harm”, like in the Swiss context where it is legally classified as “severity degree 0” (Federal Department of Home Affairs, 2022), it is obviously preferable to use (many) animals who are killed before research rather than (a few) living animals who would suffer to some extent with a corresponding severity degree > 0.

Prima facie, decision-making in animal experimentation appears consistent with a “death does not matter morally” account of animal killing.

(2) Death matters morally

The idea that animal death matters morally can be based on the argument that animals are deprived of opportunities in their future life if their life is ended. The extent to which they are aware of those opportunities or their own future is not always considered relevant (DeGrazia, 1996, 237 ff.)

Among those defending an opportunity-based account of animal death, some argue that death is the ultimate harm to animals, even if they do not have an understanding of death. As animal rights theorist Tom Regan put the point: “[A]n untimely death is a deprivation of a quite fundamental and irreversible kind. It is irreversible because, once dead, always dead. It is fundamental because death forecloses all possibilities of finding satisfaction.” (Regan, 2004) (p.100). Others suggest that the harm of death must be weighed against other harms to animals in decision-making (weighing account). The badness of death is also understood as a deprivation, in this case. While death deprives the animal of future opportunities, the prospect of life must be overall positive, to be preferable to death. This is, for example, reflected in animal quality of life assessments, including the idea of a “life not worth living” (in which case death is preferable) or a “life worth avoiding” (in which case “the balance of salient positive and negative experiences is unfavorable, but can be remedied rapidly by veterinary treatment or a change in husbandry practices”) as developed by Green and Mellor (2011). Michael Cholbi (Cholbi, 2017) accordingly suggests a weighing process to determine an optimal point for ending animals ‘ lives in the veterinary context: the opportunities for good experiences and future suffering are weighed against the certain harm of death.

Furthermore, there is an ongoing debate on (some) animals’ capacities to make plans or have desires for their future lives. These become morally relevant for, e.g., the time-relative interest account on animal death *(*McMahan, 2016*)* which includes considerations of the degree of connectedness between the animal’s current and future self. Research on different species’ capacities in this regard might lead to additional influential decision-making criteria in animal research (Shaw et al., 2014). For a more elaborate discussion of this debate, see, e.g., Selter (2020).

For all here-presented accounts that consider death as a harm, it can be argued, accordingly, that the degree of harm through death might differ for different groups of animals, depending on, e.g., the extent to which they are capable of having subjective experiences and, consequently, subjective welfare. Therefore, it could be justified to consider, for example, killing fruit flies as a lesser harm than killing mice. However, deprivation can be understood as a harm beyond subjective perception and might therefore be applied to all kinds of animals. The specifications of animal groups and differentiated harms are beyond the scope of this paper and more research is needed to clarify to what extent death as a harm applies to which part of the animal kingdom.

Comparing basic assumptions in medical research on humans and animals, Angela Martin, additionally, elaborates on the question of what animal research could look like if basic animal rights were accepted (Martin, 2022). She suggests treating nonhuman animal research subjects like other vulnerable groups who cannot consent or speak for themselves but who should not be excluded from research as they would, thereby, be excluded from the research’s potential benefit as well. While research which is not risky or harmful to animals would be allowed within that framework, killing them (in advance, during, or after the experiment) in order to reduce harm would not.

Taking the presented approaches into account, the authors encourage the ethics of laboratory animal research, in line with further application contexts of animal ethics (companion animal ethics, farm animal ethics), to shift towards a perspective of death as a harm that should be weighed in the harm-benefit analysis and considered as part of the 3R principles.

### Stakeholders’ Perspectives on Killing in Animal Experimentation

As recent literature (Nøhr et al., 2016) suggest the importance of including the perspectives of those involved in animal research practice (Blomberg, 2019; Franco & Olsson, 2014; Franco et al., 2018; Greenhough & Roe, 2018; King & Zohny, 2022; Newsome et al., 2019) (i.e., stakeholders such as researchers, animal welfare officers, veterinarians, animal technicians and caretakers, etc.), this section should give an overview of potential and actual viewpoints by those stakeholders on killing in animal experimentation. This is not meant as an exhaustive literature review but as an exploration of positions and arguments.

(a) Killed during experimentation

Cases of animals killed during experimentation can be distinguished from the other three classes in at least two ways from the animal researcher’s perspective, i.e., animal research per se is meaningful and approved studies are justified. These killings are (instrumentally) “necessary” deaths, firstly, if it is an essential part of the experiment to kill the animal. If the research question involves lethal stages or if, e.g., it is the researcher’s intention to take organs from the animal, this is incompatible with the continuation of the animal’s life. Animal researchers may perceive this kind of animal death as a necessary evil (Franco & Olsson, 2016). Within this context, secondly, killing those animals might be perceived as being necessary in a moral sense, given that, in the decisive moment, being killed is in the animal’s presumed best interest, and, therefore, preferable to “awaiting their ‘spontaneous’ death” (Franco et al., 2012, 1). Those cases of killing could, accordingly, be considered as euthanasia in a narrow sense (Persson et al., 2020), in contrast to the other three classes of animals that are killed for other reasons than ending their suffering. The way animals are killed during experiments is a common research issue and, more recently, the effect killing procedures have on laboratory staff has been explored (Roe & Greenhough, 2023). The mere act of killing laboratory animals creates stress in laboratory workers and is a risk factor to psychological strain and compassion fatigue (Newsome et al., 2019; Rumpel et al., 2023; Scotney et al., 2015). Furthermore, the challenge of correctly defining “humane endpoints” is addressed by various authors (Herrmann & Flecknell, 2018; Morton & Hau, 2021; Najafi et al., 2020). Overall, the focus is rather on details of the killing process (who, when, and how?) than on questioning the ethics of killing animals more generally.

(b) Animals killed in advance

This focus on “how” to kill research animals rather than “if at all” is very much in line with the legal focus on the prevention of animal harm (where animal death is not considered a harm), if need be by killing the animal in advance. Despite the scientific progress in alternatives to animal research, working on dead animals is still accepted as a replacement method. In part, that phenomenon points towards the necessity to differentiate between the process of replacing *live* animal experiments by (e.g., non-animal or dead animal) alternatives (see section “Cases of killing and 3R”) and the use of non-animal methods that are part of the new-approach methodology but do not replace a formerly existent animal experiment (Grimm et al., 2023).

Furthermore, the inclusion of the use of killed animals in the definition of Replacement is occasionally problematized by the scientific community (Redmond, 2019), as discussed in the section “Cases of killing and 3R”.

(c) “Surplus” animals

Both animal ethicists and laboratory animal researchers are currently discussing “surplus” animals (Chmielewska et al., 2015). “Surplus” animals are not only known in the laboratory but also from the debate on banning the killing of male chicks (BANKC) (Gremmen & Blok, 2016; Gremmen et al., 2018) and, increasingly, animals from zoos (Asa, 2016; Gunasekera, 2018) (Browning, 2018). In all cases, the problem of a large number of healthy animals who can, for economic and logistic reasons, not be kept is challenged not only by animal ethicists but also by stakeholders working in the respective sectors. From farmers and farm animal veterinarians, we learn that they consider animal death as particularly problematic if the animal’s “purpose” is not fulfilled (Fahrion et al., 2011, p. 211; Hartnack, 2022) and, in contrast, as justifiable if their human-imposed destiny—in that case, being processed to produce human food or clothes—was fulfilled (Fahrion et al., 2011). For the animal who is killed, however, the purpose of an animal’s death—as long as it is not in the animal’s (presumed) best interest—does not make a difference.

When it comes to laboratory animals, stakeholders again differentiate between avoidable deaths, i.e., animals that would not have been needed but are, e.g., bred to grant a fast supply, and unavoidable deaths, e.g., animals that do not carry the requested genetic or phenotypic properties. For an exhaustive presentation of facts and arguments see Greenough and Roe (2018). In some countries, the problem is outsourced via buying the animals from commercial breeding companies and not calculating the surplus animals who are born there as part of the research process, which could also contribute to the minimisation of surplus animals (Greenhough & Roe, 2018). Other institutions might transfer all their research abroad to circumvent stricter laws on preventing surplus animals (Wewetzer et al., 2023). At the same time, the large numbers of animals killed as “surplus” provide a very powerful opportunity for a decrease of the number of animals killed in animal research [ibid.]. The same authors, however, suggest the use of invertebrates [ibid.]—a strategy that would most likely not reduce the number of killed animals, but might decrease the overall harm through death, as it is sometimes argued if what the animal is deprived of is different for groups of animals, e.g., between vertebrates and invertebrates (Browning & Veit, 2020).

(d) “Leftover” animals

The debate is focused on practical suggestions on how to deal with “leftover” animals instead of killing them, such as reusing them in further experiments or “rehoming” them (Chmielewska et al., 2015; N. H. Franco, 2016). In contrast to the debate on the (surplus) animals’ purpose, the focus here is on the avoidance of killing at almost all costs. As for the animals killed during research, the burden killing puts on laboratory staff might play a role. However, both reuse and rehoming are only feasible for a small sub-group of the numerous “leftover” animals, and certainly, the scientific community is most aware of it. Specific recommendation for rehoming such as, e.g., in the most recent document by the FELASA (Ecuer et al. 2023) are limited to dogs and cats, despite the by far larger availability of rodents as potentially rehomed animals.^1^

## Discussion

Like in other fields of human-animal interaction (e.g., farm animals, companion animals, wildlife), taking animal lives in the laboratory is an issue that causes increasing public discomfort. Unfortunately, quantitative data on attitudes towards killing animals is exceedingly scarce, as large values surveys such as the *World Values Survey* (2022) fail to consider animal issues altogether. However, there are signs of an increasing concern with killing animals in certain societies. Those concerns are not only underlined in cases of companion animals, e.g., with a growing animal hospice movement (Shearer et al., 2017), the demand for palliative care for companion animals and converging death ideals in human and veterinary medicine (Selter et al., 2022), but also by complaints about the practice of killing “surplus” animals in zoos (Powell et al., 2018) and—particularly during the pandemic—in animal shelters (Pepper, 2023). The recent development in BANKC in Europe as well as reactions to the killing of laboratory animals when studies were interrupted during the pandemic (D. Grimm, 2020) mirror general concern about taking animal lives, even if it is executed painlessly. Reasons for the intuition that animal death matters in a morally relevant way can be manifold and they depend on the context. Mortality is something that connects us with the rest of the animal kingdom. In contrast to most of us, however, most nonhuman animals are unaware of it. End-of-life situations in companion animals, thus, occasionally resemble end-of-life situations involving human family members while arguments for decisions (not) to end animal lives can be based on animal-centred or human-centred factors. The former include the lifetime that would be left for the animal (deprivation aspect) or the suffering the animal would have to bear if it continued to live (weighing aspect) (Persson et al., 2022). The latter entail time for saying goodbye or ideals of a good death (Persson et al., 2022). The additional question if the animal has lived a full life-span (fair innings principle, (Persson et al., 2022)) can be raised in companion animal but also in farm animal contexts and might provide an explanation why it is publicly more acceptable to raise and kill the brothers of laying hen chicks than to kill them immediately, even if it is questionable if their lives as farmed roosters will be happy or meaningful.

Not least, the BANKC debate presented the initial reason to question the justification for killing surplus lab animals according to German law (Chmielewska et al., 2015; Wagenknecht et al., 2023).^2^

In laboratory animals, the above-presented arguments can be applied to the different classes of killed animals. For those killed during experimentation, the central question would be: Should the animal be killed during the experiment (and be deprived of a future life but free from suffering) or should the animal continue to live, including the suffering during and after the experiment? An ethical shift in valuing animal life might lead to interesting answers in an empirical survey on that question. For animals killed before research, the central questions would be: Is painless death considered a harm to the animal? And is painless (i.e., also free of other forms of harm, stress, fear etc.) killing indeed possible? For “surplus” and “leftover” animals, but also for the other two groups, the fair innings argument could be explored: Do these animals live a full life-span? Do they experience what it means to live the life of a mouse (or rat, or zebrafish, etc.) and might that be different for different animal species? While the public’s perspective on these questions should not alone define decision about the regulation of killing animals in research, they definitely should be considered. The public is a key stakeholder in animal research, not only benefiting from it but in most cases funding it with tax money. In this sense, to ensure the legitimacy of animal research done on behalf of the public, regulations (including those regarding the killing of research animals) should consider these perspectives.

The high attention accorded to the killing of “surplus” animals compared to those killed before or during experimentation might be due to the latter being perceived as “necessary” or “unavoidable”. Given that animal experiments should only be approved if no animal-free alternatives are available to answer the research question, this attitude might seem plausible (though only on the assumption that the research question itself is necessary to answer). However, killing before research is, as stated above, already considered an alternative because no animals are harmed (if death is not considered a harm) and no animal experiment is taking place. In other words, there is a pivotal difference between the regulation and, accordingly, demand for justification, of the use of animals that are euthanised or killed in animal experiments, and the use of animals that are killed before procedures are conducted with their bodies. Although researchers can be asked to “reuse” animals that were used in other studies before to avoid “leftovers”, there are research areas in which the type of (genetically modified) animal is so specific that the only way to obtain them is to breed them specifically for the study. There are attempts to use the network of research institutions to make animals, or their organs and other tissues, available that could be used for research elsewhere like “animatch” (AniMatch UG, 2024). The idea of rehoming is challenging in a similar way as animals can only be placed in a home outside the lab if they are not genetically modified, have not undergone a treatment that poses a danger to themselves, other animals, or the future “owners”, and if there is a demand for that animal species as a companion for humans. It is therefore extremely unlikely that the large number of rodents that are currently killed can ever be rehomed in the future. For the vast majority of animals that are bred in the context of animal research, on the one hand, being killed is the economically most efficient and, consequently, the most realistic fate, even though an ethically sound argument is lacking and phenomena like animatch and rehoming initiatives point towards an overall uncomfortable gut feeling among stakeholders concerning killing. The BANKC debate, on the other hand, suggests that if the stakes are sufficiently high—and animal “life or death” seems to be high enough—even demanding changes can legally be implemented despite economic disadvantages.

## Conclusion

Legislation and professional (e.g., veterinary) practice answered to the public and scientific debate on animal life and death by adapting to the changing concerns of society. It is time that animal welfare laws regulating animal experimentation take the killing of animals into consideration. As a globally acknowledged principle, the 3Rs should be discussed in this respect. As a framework advocating for an incremental improvement of the animal experimental practice, though, it is difficult for the 3Rs to cover an “all-or-nothing” procedure like killing. While it is possible to refine the “how”, there is no stepwise approach to the “if” of killing—a laboratory animal either survives or it is killed. With the shift from a mainly welfarist view on animals towards an attitude that perceives animals’ lives as worthy of protection, animal experimentation should include measures and corresponding guidelines to at least reduce the number of animals (of all groups) who are killed for research purposes. The continuing discourse on the moral status and, accordingly, the treatment of nonhuman animals might eventually lead to the (animal rights based) attitude that only such research that animals would agree to be a part of would be morally acceptable (Martin, 2022). Most likely, that would exclude experiments that involve killing of research participants.

In the meantime, we suggest possible reformative approaches of the 3Rs, for example in a way that:

- They make the underlying philosophical position transparent. We suggest it should be based on a weighing account of animal death, which is in line with the treatment of animals in other contexts.
- They are applicable to procedures on living and dead animals, which leads to the next aspect
- The principle of reduction is applied to procedures on dead animals, too, thus demanding the attempt to decrease the number of killed animals for research projects
- The principle of replacement entails that methods using (parts of) dead animals need to be replaced by animal free methods, if possible; and replacing research on living animals by research on killed animals is not considered a goal;
- They comprise all kinds of animals because arbitrary boundaries such as being vertebrates do not matter to the accounts on animal death; a differentiation between the severity of harm for different animal groups would need to be elaborated;
- They are applied to the broader context of experimentation, including breeding and the fate of the animals after the experiment, demanding that “surplus” and “leftover” animals cannot be killed if it is not in their presumed best interest. If the 3Rs remain restricted to the context of the experiment only, a different framework such as PREPARE (Norecopa, 2024) should regulate animal death in the broader context.

Questions concerning the extent to which these claims are feasible or the infrastructure that would be needed to implement the suggested changes call for more empirical research on stakeholder perspectives in this context. What, for example, would it mean for decision-makers if surplus animals could not be killed? What if researchers were no longer allowed to conduct experiments on dead animals without a research proposal? How would the harm of death be weighed against the scientific benefits of a given experiment? Confronting stakeholders with those questions may reveal valuable insights for future “refinement” of the 3Rs.

## Footnotes

1 Moreover, the increasing use of genetically-modified animals represents an additional challenge for the reuse or rehoming of these animals, as they cannot legally be released from their facilities.

2 However, potential reasons and consequences regarding this increase in public concern need to be considered. With an ambivalent attitude towards human death and, at the same time, a close relationship between many companion animals and their “owners”, concerns might not only be raised due to the animal losing their life but also due to the human who is losing a part of their family. In the case of BANCK, and similarly in the case of male dairy calves, the alternative prospect of living a short life full of deprivation as a broiler chicken or a beef cow might in the end not be preferable from the animal’s perspective to a quick and painless death. Accordingly, the public attention that is given to the protection of animal life needs to be analysed carefully. As there is a strong link between the preceding BANKC and the debate on killing laboratory animals, a more thorough look at the public opinion in the BANKC debate might shed light on potential public arguments on killing animals in the laboratory.
